# Depression May Not Be Related to Impaired Interoceptive Sensibility: The Role of Alexithymia

**DOI:** 10.3390/bs15080995

**Published:** 2025-07-22

**Authors:** Haoran Shen, Juanhua Li, Renjie Zeng, Yaping He, Jingang Dai, Zezhi Li, Youfa Li, Gaoxia Wei

**Affiliations:** 1 Collaborative Innovation Center of Assessment for Basic Education Quality, Beijing Normal University, Beijing 100875, China; shr84728758@163.com; 2 Department of Psychiatry, The Affiliated Brain Hospital of Guangzhou Medical University, Guangzhou 510370, China; jane320000@21cn.com; 3 State Key Laboratory of Cognitive Science and Mental Health, Institute of Psychology, Chinese Academy of Sciences, Beijing 100101, China; zrj7758258@gmail.com; 4 Department of Psychology, University of Chinese Academy of Sciences, Beijing 101408, China; 5 School of Chemistry and Chemical Engineering, Henan Normal University, Xinyang 453007, China; heyaping0203@163.com; 6 National Chinese Medicine Experts Inheritance Office, Experimental Research Center, China Academy of Chinese Medical Sciences, Beijing 100700, China; zhongyidai@163.com

**Keywords:** impaired interoception, interoceptive sensibility, MAIA, heartbeat counting task, alexithymia, alexithymic depressed patients, depression, heterogeneity, mediation, emotional disorders

## Abstract

Interoceptive impairments are increasingly recognized as psychopathology processes underlying emotional disorders. However, their relationship with depression remains inconclusive. Alexithymia may influence the association between interoception and depressive symptoms. This study aimed to examine the role of alexithymia between interoception and depression. Eighty-eight depressed patients (DEPs) and fifty healthy controls (HCs) were recruited. Interoceptive sensibility was assessed using the Multidimensional Assessment of Interoceptive Awareness, and interoceptive accuracy and interoceptive awareness were evaluated through heartbeat counting tasks. Alexithymia was measured with the Toronto Alexithymia Scale. In the DEP group, depressive symptoms were assessed using the Hamilton Depression Scale. In DEPs, none of the three dimensions of interoception were associated with depressive symptoms. The alexithymic depressed patients exhibited lower interoceptive sensibility than their non-alexithymic counterparts, while the latter did not differ from the HC group. Moreover, alexithymia mediated the link between interoceptive sensibility and depressive symptoms. These results suggested that impaired interoceptive sensibility may primarily contribute to alexithymia, which, in turn, leads to depression. This highlights the importance of addressing alexithymia in therapeutic interventions aimed at improving the interoceptive process in depressed individuals.

## 1. Background

Depression is a disorder of allostasis, which is characterized by abnormalities in neurologic, metabolic, and immunologic systems, as well as pervasive negative affect (Barrett et al., 2016). Abnormal afferent sensory inputs from within the body, namely disturbed interoception, have been considered as crucial for the psychopathology and psychosomatic process of depression (Eggart et al., 2019; Zhou et al., 2024). Interoception is defined as the process by which the nervous system senses, interprets, and integrates signals originating from the body (Khalsa et al., 2018). This multi-dimensional process encompasses interoceptive sensibility, interoceptive accuracy, and interoceptive awareness (Garfinkel & Critchley, 2013). Interoceptive sensibility refers to the subjective perception and beliefs about one’s internal physical state. Interoceptive accuracy pertains to the objective ability to detect bodily signals, such as performance on tasks like heartbeat counting or heart discrimination. Interoceptive awareness involves a meta-cognitive understanding of one’s interoceptive experiences (Garfinkel & Critchley, 2013).

Several studies have identified interoceptive impairments among depressed patients (Datko et al., 2022; Davey, 2025; Dunn et al., 2007). For instance, clinically depressed individuals often exhibit dysfunction in interoceptive sensibility, as reflected by lower scores on self-reported measures such as the Multidimensional Assessment of Interoceptive Awareness (MAIA) and the Body Perception Questionnaire (BPQ) (Avery et al., 2014; Datko et al., 2022; Fissler et al., 2016). Additionally, compared to the healthy control group, depressed individuals demonstrate reduced accuracy in tasks assessing interoceptive abilities, such as heart discrimination (Bär et al., 2012). Other studies, however, have yielded different findings (Desmedt et al., 2022). For instance, one study reported no significant differences in interoceptive sensibility between moderately and severely depressed individuals, nor any considerable association between interoceptive sensibility and depressive symptoms (Dunn et al., 2007). Similarly, a meta-analysis investigating interoceptive accuracy in major depressive disorders identified an inverted U-shaped relationship: individuals with moderate depression demonstrated more pronounced interoceptive impairments than those with slight or severe depression (Eggart et al., 2019). Moreover, no significant correlations were found between interoceptive accuracy and depressive symptoms in patients with major depressive disorders (Furman et al., 2013).

The inconsistent results may be due to the fact that depression encompasses a wide range of heterogeneous symptoms and often co-occurs with other conditions (Chai et al., 2023). Alexithymia is defined as a personality trait characterized by difficulties in identifying and describing feelings, an externally oriented thinking style, and a reduced capacity for fantasy (Sifneos, 1973). Research has shown that alexithymia is highly prevalent among individuals with depression. The prevalence of alexithymia in clinically depressed patients ranges from 27% (Leweke et al., 2012) to 50% (Kim et al., 2008), compared to approximately 10% in the general population (Franz et al., 2008). Research has shown that depressed patients with alexithymia tend to experience more severe depressive symptoms (Celikel et al., 2010; Taycan et al., 2017). In addition, alexithymic depressed patients are less likely to benefit from psychotherapy (Guenther et al., 2016) and often require higher doses of antidepressant medications compared to their non-alexithymic counterparts (Serafini et al., 2020). This suggests that alexithymia might influence the relationship between interoception and depression.

On the other hand, accumulative evidence has shown that interoceptive deficits are closely associated with alexithymia (Brewer et al., 2016; Van Bael et al., 2024). A negative relationship between interoception and alexithymia has been consistently confirmed in psychiatric disorders, primarily in autism spectrum disorders and eating disorders (Butera et al., 2023; Datko et al., 2022; Westwood et al., 2025). Research indicated that alexithymia was more prevalent among individuals with diminished interoceptive abilities (Bornemann & Singer, 2017; Scarpazza et al., 2022). While some researchers have hypothesized that alexithymia in depressed patients might be linked to reduced overall bodily awareness (Bär et al., 2012; Herbert et al., 2007), few studies have directly investigated this connection in depressed individuals. Moreover, existing research on the interplay between interoception and alexithymia remains limited and has often yielded contradictory findings across different populations. For example, one study reported a strong correlation between impaired interoception and high levels of alexithymia in both general and psychiatric populations, including individuals with depression (Brewer et al., 2016). In contrast, a meta-analysis found that interoception was negatively correlated with alexithymia exclusively in clinical populations, with no significant relationship observed in the general population (Trevisan et al., 2019). This leaves open the question of whether the association between interoception and alexithymia in depression exhibits a unique pattern distinct from that of the general population.

Therefore, further investigation is warranted to examine whether all patients with depression exhibit impaired interoception. And does this relationship depend on alexithymia? This study aimed to examine the role of alexithymia in interoception and depression by comparing the depressed patient (DEP) group and the healthy control (HC) group. We hypothesized the following: (1) Within the DEP group, interoception would not be significantly associated with depressive symptoms. (2) Previous studies have suggested that alexithymia in HC groups is primarily linked to psychological trauma or other environmental factors, while in patients, alexithymia is driven by the abnormal signaling of the inner body and atypical interoception (Trevisan et al., 2019). Consistently with this view, we hypothesized that a negative association between interoception and alexithymia would be observed exclusively in the DEP group, while no such association would emerge in the HC group. (3) Within the DEP group, alexithymia would partially mediate the relationship between interoception and depressive symptoms.

## 2. Method

### 2.1. Participants

The sample size was calculated using the GPower 3.1 program (Faul et al., 2007), conducting a priori power analysis for the *F* test family using regression. With an alpha level of 0.05, an effect size of 0.30, and a desired power of 0.80, the analysis indicated that 34 participants per group were required. Finally, a total of 138 Han Chinese participants were included in this study in May 2024, including 88 depressed patients (DEPs) and 50 healthy controls (HCs). The DEP group was recruited from the Affiliated Brain Hospital of Guangzhou Medical University (Guangzhou Huiai Hospital). Participants were identified through routine clinical evaluations of individuals presenting to the hospital’s inpatient psychiatric departments. Diagnoses and clinical characteristics were assessed by trained psychiatrists with at least five years of clinical experience using the International Classification of Diseases-10 (ICD-10). The inclusion criteria for the DEP group were as follows: (1) adherence to the ICD-10 diagnostic criteria for major depression; (2) an age of 18–50 years; (3) no co-occurring mental health disorders other than depression; (3) no serious chronic somatic diseases; (4) no history of substance dependence or abuse. The HC group was recruited through an online procedure. Participants were invited to take part through advertisements posted on reputable online platforms to ensure wide accessibility. Interested individuals were asked to complete a brief online screening questionnaire to assess eligibility based on the following criteria: (1) an age between 18 and 50 years and (2) no history of mental health disorders or major medical conditions. Written informed consent was obtained from all participants before this study.

This study was approved by the Independent Ethics Committee of the Guangzhou Huiai Hospital. The study adhered to the ethical guidelines of the 1975 Declaration of Helsinki. The research was registered with the China Clinical Trials Registry (CCTR) under the registration number ChiCTR2400080998, with a registration date of 20 February 2024.

### 2.2. Measurements

#### 2.2.1. Interoception

Three metrics of interoception were examined: interoceptive sensibility, interoceptive accuracy, and interoceptive awareness. Interoceptive sensibility was measured by the Multidimensional Assessment of Interoceptive Awareness (MAIA) (e.g., “When I am tense, I know where the tension is located in my body”; “I notice how my body changes when I am angry”) (Mehling et al., 2012). The MAIA consists of 32 items in 8 subscales, including noticing, not-distracting, not-worrying, attention regulation, emotional awareness, self-regulation, body listening, and trust. All items were scored on a 6-point scale, ranging from 0 (never) to 5 (always). Higher scores represent higher interoceptive sensibility. The MAIA has been translated into Chinese and shown acceptable reliability and validity (Cronbach’s alpha = 0.91; composite validity = 0.87) (Lin et al., 2017).

Interoceptive accuracy was evaluated by a heartbeat counting task (Schandry, 1981). In this task, participants were instructed to silently count their heartbeats over intervals of 25 s, 35 s, and 45 s, with each interval repeated twice in a random order. Participants were explicitly instructed not to take their pulse or use any other means to assist in counting. The counted heartbeats were then compared with actual heartbeats recorded by a heart rate monitor, and interoceptive accuracy scores were calculated using the following formula:

Interoceptive accuracy scores = 1/6 Σ (1 − (|recorded heartbeats − counted heartbeats|)/recorded heartbeats).

After each heartbeat counting trial, participants rated their confidence in the accuracy of their heartbeat estimate on a Likert scale from 0 (“Not at all confident”) to 10 (“Completely confident”). Interoceptive awareness scores were calculated as the Pearson correlation between accuracy and confidence ratings across the 6 trials (Garfinkel et al., 2015).

#### 2.2.2. Alexithymia

The 20-item Toronto Alexithymia Scale (TAS) was used to assess alexithymia in both the DEP and HC groups. The TAS includes 20 items divided into 3 subscales: difficulty identifying feelings (e.g., “I am often confused about what emotion I am feeling”), difficulty describing feelings (e.g., “I am able to describe my feelings easily”), and externally oriented thoughts (e.g., “I prefer talking to people about their daily activities rather than their feelings”). Each item is rated on a 5-point scale, with responses ranging from 1 (strongly disagree) to 5 (strongly agree), where higher scores indicate more severe alexithymia. A total greater than 60 denotes significant alexithymia (Bagby et al., 2020; Taylor et al., 1997). The Chinese version of the TAS-20 has been validated in previous studies, demonstrating good reliability and validity for use with Chinese populations (Cronbach’s alpha = 0.83; test–retest reliability = 0.87) (Jinyao et al., 2003).

#### 2.2.3. Depressive Symptoms

The Hamilton Depression (HAMD) scale was used to assess depressive symptoms in the DEP group. The HAMD scale consists of 17 items that evaluate a range of depressive symptoms, including mood, guilt, insomnia, anxiety, and somatic complaints. Each item is rated on a 5-point scale, with scores ranging from 0 (absent) to 4 (severe), where higher scores indicate more severe depressive symptoms (Hamilton, 1960). The assessments were conducted by psychiatrists with a minimum of five years of clinical experience, all of whom underwent training in administering the HAMD assessment. The scale has been demonstrated to have great reliability and validity in the Chinese population, with an interrater reliability of 0.91 (Zheng et al., 1988).

### 2.3. Procedure

The DEP group was recruited in person and completed the MAIA, heartbeat counting task, TAS, and HAMD assessments. The HC group was recruited online, with all participants completing an online questionnaire that included the MAIA and TAS. Both the DEP and HC groups were assessed within the same timeframe.

### 2.4. Statistical Analysis

All data were analyzed using SPSS 26.0 (IBM Corp., Armonk, NY, USA). First, the Kolmogorov–Smirnov test was applied to assess the normality of data distribution. Cronbach’s alpha was calculated to evaluate the internal consistency of the MAIA, TAS, and HAMD scale. Next, Pearson correlation analysis and general linear models were employed to analyze the associations between three dimensions of interoception and depressive symptoms within the DEP group. Then, using a TAS cutoff score > 60 to indicate significant alexithymia (Taylor et al., 1997), it was found that the prevalence of alexithymia was 38.64% in the DEP group and 14.00% in the HC group. Thus, the DEP group was subsequently divided into two subgroups based on TAS scores, which created an alexithymic depressed (A-DEP) group and a non-alexithymic depressed (NA-DEP) group. Differences in alexithymia and interoception among the A-DEP, NA-DEP, and HC groups were then compared using one-way ANOVA. General linear models were employed to further explore the associations between interoception and alexithymia separately for the DEP and HC groups. Finally, mediation analyses were performed to evaluate the mediating role of alexithymia between interoception and depressive symptoms within the DEP group. The mediation analysis was conducted using the PROCESS macro for SPSS (version 3.5) developed by Hayes. Indirect effects were assessed using bias-corrected confidence intervals derived from 5000 bootstrap samples, with statistical significance indicated when the 95% bootstrap confidence interval excluded zero. Standardized coefficients (β) were reported. To control for multiple comparisons, the Benjamini–Hochberg false discovery rate (FDR) was applied according to previous studies (Benjamini & Hochberg, 1995; Benjamini & Yekutieli, 2001). All statistical tests were two-tailed, and *p* < 0.05 indicated statistical significance.

## 3. Results

### 3.1. Associations Between Interoception and Depressive Symptoms in the DEP Group

The DEP group consisted of 21 males and 67 females. The mean age of the participants was 25.81 ± 8.12 years, with an age range of 19 to 48 years. The mean illness duration was 3.13 ± 2.79 years, ranging from 1 to 11 years.

The Kolmogorov–Smirnov test indicated that all the measures followed a normal distribution (*p* > 0.05). The Cronbach alpha values were 0.91 for the MAIA, 0.86 for the TAS, and 0.88 for the HAMD, indicating that the reliability of these measures was acceptable for this study.

Pearson correlation was conducted to examine the associations between different variables in the DEP group. The results indicated that neither interoceptive sensibility (r = −0.18; *p* = 0.133), interoceptive accuracy (r = −0.19; *p* = 0.157), nor interoceptive awareness (r = −0.18; *p* = 0.112) was significantly associated with depressive symptoms. (See Table 1).

The general linear models were further utilized to analyze these associations. The normality test of residuals found that the residuals were normally distributed (*p* > 0.05). The analysis revealed that none of the three dimensions of interoception—namely, interoceptive sensibility scores (β = −0.26; 95% CI: −0.59; 0.08; $R^{2}$ = 0.02), interoceptive accuracy scores (β = −0.29; 95% CI: −0.15; 0.03; $R^{2}$ = 0.07), nor interoceptive awareness scores (β = −0.46; 95% CI: −2.28; 1.36; $R^{2}$ = 0.11)—were associated with depressive symptoms.

### 3.2. Comparison of Interoception Among A-DEP, NA-DEP, and HC Groups

**Figure 1:**
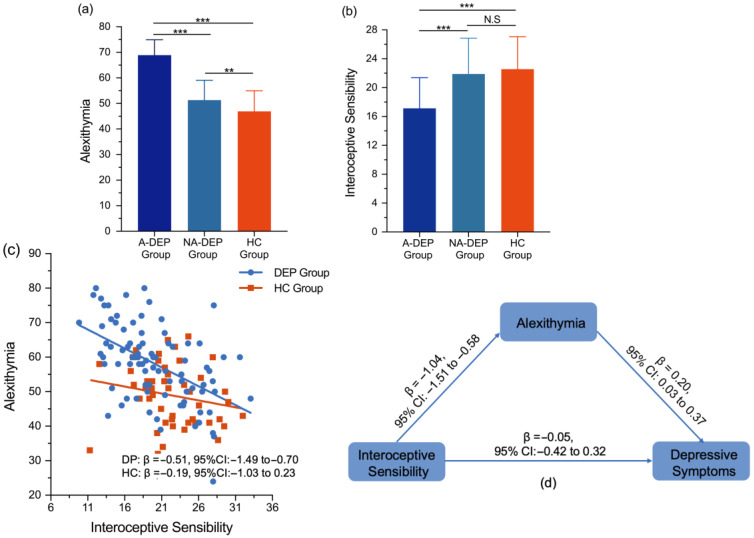
(**a**) Group differences in alexithymia in the A-DEP, NA-DEP, and HC groups; (**b**) group differences in interoceptive sensibility in the A-DEP, NA-DEP, and HC groups; (**c**) the linear models in the DEP and HC groups; (**d**) the mediating role of alexithymia between interoceptive sensibility and depressive symptoms in the DEP group. Abbreviations: A-DEP Group, alexithymic depressed patient group; NA-DEP Group, non-alexithymic depressed patient group; HC group, healthy control group; DEP Group, depressed patient group. Notes: *** *p* < 0.001; ** *p* < 0.01; N.S, non-significant; β, standardized coefficient.

Depressed patients were divided into A-DEPs and NA-DEPs according to alexithymic scores. A-DEPs showed significantly higher alexithymic scores than NA-DEPs (F = 127.78; *p* < 0.001; $\eta^{2}$ = 0.60). Interoception differences were compared among the A-DEP, NA-DEP, and HC groups. The HC group consisted of 12 males and 30 females. The mean age of the participants was 26.40 ± 4.71 years, with an age range of 21 to 45 years. As shown in Table 2, there were no significant differences in age (F = 1.68; *p* = 0.372; $\eta^{2}$ = 0.04) and sex (χ2 = 0.33; *p* = 0.849; Cramér’s V = 0.04) among the A-DEP, NA-DEP, and HC groups. One-way ANOVA results indicated significant differences in alexithymia scores among the A-DEP, NA-DEP, and HC groups (F = 91.13; *p* < 0.001; $\eta^{2}$ = 0.59). Pairwise comparisons showed that the A-DEP group had higher alexithymia scores than both the NA-DEP (*p* < 0.001) and HC groups (*p* < 0.001), and the NA-DEP group scored higher than the HC group (*p* < 0.01). After applying FDR correction, these differences remained significant (*p* < 0.01) (Table 2, Figure 1a).

For interoceptive sensibility, significant group differences were also observed across the A-DEP, NA-DEP, and HC groups (F = 15.18; *p* < 0.001; $\eta^{2}$ = 0.19). Pairwise comparisons indicated that the A-DEP group scored lower in interoceptive sensibility than both the NA-DEP (*p* < 0.001) and HC groups (*p* < 0.001), while no significant differences were observed between the NA-DEP and HC groups (*p* = 0.48) (Table 2, Figure 1b). After applying FDR correction, these significant differences remained significant (*p* < 0.01).

In the heart counting task, the A-DEP group showed marginally lower scores in interoceptive accuracy compared to the NA-DEP group (F = 3.70; *p* = 0.058; $\eta^{2}$ = 0.16), while differences in interoceptive awareness were not statistically significant (F = 2.68; *p* = 0.106; $\eta^{2}$ = 0.11) (Table 2). After FDR correction, the marginally significant differences in interoceptive accuracy became non-significant.

### 3.3. Associations Between Interoception and Alexithymia in the DEP and HC Groups

In the DEP group, the general linear model revealed that interoceptive sensibility scores (β = −0.51; 95% CI: −1.49; −0.70; $R^{2}$ = 0.26) were negatively associated with alexithymia scores, while interoceptive accuracy scores (β = −0.10; 95% CI: −3.55; 1.45; $R^{2}$ = 0.01) and interoceptive awareness scores (β = −0.05; 95% CI: −2.63; 2.52; $R^{2}$ = 0.09) were not related to alexithymia scores (Figure 1c).

However, in the HC group, no significant association was found between interoceptive sensibility scores and alexithymia scores (β = −0.19; 95% CI: −1.03 to 0.23; $R^{2}$ = 0.01) (Figure 1c).

### 3.4. Mediation Effects of Alexithymia Between Interoception and Depressive Symptoms in the DEP Group

Findings revealed that alexithymia scores partially mediated the relationship between interoceptive sensibility scores and depressive symptoms. Specifically, the mediating effect of alexithymia scores on the relationship between interoceptive sensibility scores and depressive symptoms was significant (β = −0.21; 95% CI: −0.39 to −0.02), whereas the direct effect of interoceptive sensibility scores on depressive symptoms was not statistically significant (β = −0.05; 95% CI: −0.42 to 0.32) (Table 3, Figure 1d). To further examine the mediating role of alexithymia between interoceptive sensibility and depression, the eight subscales of interoceptive sensibility were included in the mediation models. As shown in Table 3, alexithymia mediated the associations between not-worrying (β = −0.66; 95% CI: −1.43 to −0.04), attention regulation (β = −0.78; 95% CI: −1.74 to −0.11), and body listening (β = −0.73; 95% CI: −1.59 to −0.05) with depressive symptoms.

However, alexithymia scores did not significantly mediate the relationship between interoceptive accuracy scores and depressive symptoms (β = 0.01; 95% CI: −0.57 to 0.78) or between interoceptive awareness scores and depressive symptoms (β = −0.91; 95% CI: −3.95 to 0.39) (Table 3).

## 4. Discussion

The present study revealed that in the DEP group, none of the three dimensions of interoception were associated with depressive symptoms, which indicated that interoceptive deficits may not directly lead to depression. Moreover, we found that the alexithymic DEP group exhibited significantly lower self-reported interoceptive sensibility compared to their non-alexithymic counterparts, while the latter did not differ significantly from the HC group. Negative association between interoceptive sensibility and alexithymia was observed exclusively in depressed patients, with alexithymia partially mediating the relationships between interoceptive sensibility and depressive symptoms. Additionally, the mediating role of alexithymia between objectively measured interoception (interoceptive accuracy and interoceptive awareness) and depressive symptoms was not significant. These findings indicate that alexithymia, rather than impaired interoceptive sensibility, may be a more significant contributing factor to depression.

In this study, in the DEP group, no correlations were found between the three dimensions of interoception and depressive symptoms, which aligned with previous studies. For example, a review of interoception in patients with a clinically diagnosed major depressive disorder concluded that moderately depressed individuals experienced greater interoceptive impairments than those who were slightly and severely depressed (Eggart et al., 2019). Another study, which included a large sample of clinically DEPs (N = 1622), similarly reported no significant associations between interoception and depressive symptoms (Desmedt et al., 2022). Additionally, in this study, decreased interoceptive sensibility was observed only in DEPs with alexithymia, whereas interoceptive sensibility in DEPs without alexithymia was comparable to that of the general population. These findings indicated that interoceptive deficits may not directly contribute to depression, but rather, alexithymia might play a more significant role. Mediation analyses further supported this interpretation.

In the DEP group, alexithymia partially mediated the relationship between interoceptive sensibility and depressive symptoms, while the direct effect of interoceptive sensibility on depressive symptoms was not significant. In addition, the indirect effects were also significant between not-worrying, attention regulation, and body listening with depressive symptoms. These mediating roles of alexithymia have also been observed in other contexts linking interoception and maladaptive behaviors. For instance, one study found that a subdimension of the TAS—specifically, difficulty identifying feelings—mediated the relationship between interoceptive sensibility and alcohol consumption (Betka et al., 2018). Similarly, in individuals with autism spectrum disorder, alexithymia was reported to mediate the connection between interoceptive awareness and empathy (Mul et al., 2018). Desdentado et al. (2022) found that in healthy individuals, alexithymia mediated the associations between the not-worrying sub-dimension and depression. Our findings further suggested a similar association in DEPs, where abnormal awareness of internal bodily sensations may correlate with high levels of alexithymia, potentially contributing to more pronounced depressive symptoms.

Interoception is widely acknowledged to play a crucial role in emotional processing. Embodied theory posits that both the emotional experience and regulation are influenced by the arousal and feedback mechanisms from the internal bodily states. This internal awareness is essential for monitoring and interpreting subjective emotional states, including physical sensations (Schachter & Singer, 1962; Valins, 1966). As a result, individuals with interoceptive impairments tend to have difficulties in understanding or describing their emotions, namely alexithymia (Critchley, 2005; Herbert et al., 2011; Zamariola et al., 2019). On the other hand, alexithymic patients are more likely to exhibit severe depressive symptoms compared to their non-alexithymic counterparts (Leweke et al., 2012; Li et al., 2015). Individuals with high alexithymia often adopt escape–avoidance coping strategies and rely on less social support. One study found that this behavior might lead to a lack of social interactions and reduce access to emotional or practical social support from others, further exacerbating depressive symptoms (Li et al., 2015). This study also had important clinical implications for treating alexithymia in DEPs through interoceptive perspectives. Research has shown that alexithymia can be alleviated through targeted interventions (Porcelli et al., 2011). For example, a meta-analysis demonstrated that mindfulness-based interventions significantly reduced alexithymia (Norman et al., 2019). These interventions focus on cultivating present-moment awareness, particularly of bodily sensations (Bornemann & Singer, 2017), and have been shown to effectively enhance interoceptive abilities (Chen et al., 2021; Mehling et al., 2018; Shen et al., 2023). Given the strong relationship between interoception and alexithymia in depressed individuals, it is speculated that increased bodily awareness may serve as a key mechanism by which mindfulness-based intervention alleviates alexithymia (Luminet & Nielson, 2025). Desdentado et al. (2022) suggested that the interoceptive process associated with alexithymia and subsequent depression includes worrying about and ignoring uncomfortable bodily sensations, lacking trust in bodily signals, and an inability to voluntarily focus on them. Therefore, therapeutic efforts targeting alexithymic depressed patients could focus on improving trust in and attention to bodily signals and mitigating concerns about their meaning.

There were some limitations in this study. First, the sample size for both the DEP and HC groups was relatively small, which may necessitate caution when interpreting the results. Second, the questionnaires for healthy participants were administered online, preventing the assessment of interoceptive accuracy and interoceptive awareness in this group. Third, although alexithymia mediated the associations between interoceptive sensibility and depression, the self-reported measure of interoceptive sensibility (MAIA) was susceptible to biases. Conversely, no significant relationship was found between interoceptive accuracy or interoceptive awareness, as measured by the heartbeat counting task, and alexithymia. This lack of significance may be partly due to the failure to control for potential confounders, such as time perception, body mass index, knowledge about heart rate, and anxiety. Previous studies have shown that interoceptive accuracy is significantly related to alexithymia when confounders are accounted for (Murphy et al., 2018). Future studies should use objective methods to measure interoception and control for potential confounders. Fourth, the assessment of alexithymia also relied on self-report measures. Alternative assessment methods, such as the Toronto Structured Interview for Alexithymia (Bagby et al., 2006), could be employed to provide a more objective measure of alexithymia. Fifth, the association between interoception and depression is complicated, and other factors should be considered. For example, Vivas-Rivas et al. (2024) found that anxiety mediates the relationship between interoception and depression, highlighting the importance of additional psychological variables in this association. Finally, the cross-sectional design of this study precludes any conclusions about causality between interoception and alexithymia. To better understand this relationship, future studies should adopt longitudinal designs or include intervention-based approaches to explore causal pathways.

## 5. Conclusions

The present study revealed that self-reported interoceptive sensibility was impaired only in depressed individuals with high levels of alexithymia, and alexithymia partially mediated the association between interoceptive sensibility and depressive symptoms. The findings suggested that deficits in interoceptive sensibility may primarily contribute to the development of alexithymia, which, in turn, leads to depression. These results provide a new insight that depression with alexithymia might have different psychopathological mechanisms, which highlights the importance of addressing alexithymia in therapeutic interventions targeting the enhancement of the interoceptive process in depressed patients.

## Figures and Tables

**Table 1:** Pearson correlation matrix of the DEP group.

	Interoceptive Sensibility	Interoceptive Accuracy	Interoceptive Awareness	Alexithymia	Depressive Symptoms
Interoceptive Sensibility					
Interoceptive Accuracy	0.04				
Interoceptive Awareness	0.13	0.52 **			
Alexithymia	−0.51 **	−0.10	−0.31 **		
Depressive Symptoms	−0.18	−0.19	−0.18	0.33 **	

Notes: ** *p* < 0.01.

**Table 2:** Group differences among the A-DEP, NA-DEP, and HC groups.

	A-DEP Group (*n* = 34)	NA-DEP Group (*n* = 54)	HC Group (*n* = 50)	F/χ2	*p*	Effect Size
Age (Years, Mean ± SD)	25.68 ± 6.36	27.15 ± 8.84	26.40 ± 4.71	1.68	0.372	0.04
Sex (M/F)	7/27	14/40	12/38	0.33	0.849	0.04
Illness duration (Years)	3.23 ± 2.64	3.07 ± 2.90		0.17	0.685	0.02
Alexithymia	68.88 ± 6.02	51.30 ± 7.70	46.88 ± 8.07	91.13	<0.001	0.59
Interoceptive Sensibility	17.14 ± 4.25	21.90 ± 4.96	22.56 ± 4.50	15.18	<0.001	0.19
Interoceptive Accuracy	0.46 ± 0.31	0.58 ± 0.20		3.70	0.058	0.16
Interoceptive Awareness	0.25 ± 0.25	0.35 ± 0.24		2.68	0.106	0.11
Depressive Symptoms	13.73 ± 7.70	10.42 ± 6.42		4.89	0.031	0.18

Abbreviations: A-DEP Group, alexithymic depressed patient group; NA-DEP Group, non-alexithymic depressed patient group; HC group, healthy control group. Notes: The effect size for sex was measured using Cramér’s V, while for all other variables, the effect size was calculated using *η*^2^.

**Table 3:** The mediation effects of alexithymia between interoception and depressive symptoms.

Independent Variable	Indirect Effect		Direct Effect		Total Effect	
	β	95%CI	β	95%CI	β	95%CI
Interoceptive sensibility	−0.21	[−0.39, −0.02]	−0.05	[−0.42, 0.32]	−0.26	[−0.59, −0.08]
Noticing	0.59	[−0.04, 1.38]	0.61	[−1.19, 2.40]	1.20	[−0.62, 3.00]
Not-distracting	−0.33	[−0.93, 0.37]	1.08	[−0.70, 2.83]	0.75	[−1.07, 2.58]
Not-worrying	−0.66	[−1.43, −0.04]	−1.44	[−3.31, 0.43]	−2.10	[−3.92, −0.29]
Attention regulation	−0.78	[−1.74, −0.11]	−0.65	[−2.40, 1.09]	−1.43	[−3.09, −0.23]
Emotional awareness	−0.43	[−1.14, 0.10]	0.60	[−0.97, 2.18]	0.17	[−1.45, 1.81]
Self-regulation	−0.87	[−1.69, 0.03]	−0.52	[−2.17, 1.13]	−1.39	[−2.86, 0.08]
Body listening	−0.73	[−1.59, −0.05]	−0.14	[−1.70, 1.43]	−0.87	[−2.38, −0.66]
Trust	−0.84	[−2.02, 0.05]	−0.73	[−2.35, 0.90]	−1.57	[−2.93, −0.19]
Interoceptive accuracy	0.01	[−0.57, 0.78]	−2.30	[−0.41, 0.05]	−2.20	[−4.15, −0.43]
Interoceptive awareness	−0.91	[−3.95, 0.39]	−1.57	[−3.39, 0.25]	−2.48	[−3.81, −0.19]

Notes: β, standardized coefficient; 95% CI, 95% bootstrap confidence interval.

## Data Availability

Data available on request due to restrictions.
